# Evolutionary tinkering vs. rational engineering in the times of synthetic biology

**DOI:** 10.1186/s40504-018-0086-x

**Published:** 2018-08-12

**Authors:** Víctor de Lorenzo

**Affiliations:** Centro Nacional de Biotecnología-CSIC, Campus de Cantoblanco, 28049 Madrid, Spain

**Keywords:** Genetic engineering, Synthetic biology, Minimum systems, Parts, Devices, Modules, Systems, Teleology, Teleonomy

## Abstract

Synthetic biology is not only a contemporary reformulation of the recombinant DNA technologies of the last 30 years, combined with descriptive language imported from electrical and industrial engineering. It is also a new way to interpret living systems and a statement of intent for the use and reprogramming of biological objects for human benefit. In this context, the notion of designer biology is often presented as opposed to natural selection following the powerful rationale formulated by François Jacob on *evolution-as-tinkering*. The onset of synthetic biology opens a different perspective by leaving aside the question about the evolutionary origin of biological phenomena and focusing instead on the relational logic and the material properties of the corresponding components that make biological system work as they do. Once a functional challenge arises, the solution space for the problem is not homogeneous but it has attractors that can be accessed either through random exploration (as evolution does) or rational design (as engineers do). Although these two paths (i.e. evolution and engineering) are essentially different, they can lead to solutions to specific mechanistic bottlenecks that frequently coincide or converge—and one can easily help to understand and improve the other. Alas, productive discussions on these matters are often contaminated by ideological preconceptions that prevent adoption of the engineering metaphor to understand and ultimately reshape living systems—as ambitioned by synthetic biology. Yet, some possible ways to overcome the impasse are feasible. In parallel to Monod’s *evolutionary* paradox of *teleo-logy* (finality/purpose) vs. *teleo-nomy* (appearance of finality/purpose), a *mechanistic* paradox could be entertained between *techno-logy* (rational engineering) vs *techno-nomy* (appearance of rational engineering), all for the sake of understanding the relational logic that enables live systems to function as physico-chemical entities in time and space. This article thus proposes a radical vision of synthetic biology through the lens of the engineering metaphor.

## Introduction

Since the beginning of the millennium, biology has been undergoing an accelerated transition from a predominantly descriptive science to a quantitative discipline.^1^ This process began with Schrödinger’s famed book, *What is life?* written at the end of World War II (Schrödinger, 1944) which for the first time rigorously approached biological systems as entities subject to the same laws of physics as the rest of the material world. The climax of this new vision came 50 years ago with the deciphering of the structure of DNA, the genetic code, and the elements involved in the flow of information from DNA to proteins. Paradoxically, however, the role of physicists in the birth of molecular biology did not culminate in a quantitative culture or in the precise, standardized descriptive language characteristic of the hard sciences. On the contrary, molecular genetics and the molecular biology derived from it did not, with very few exceptions, take advantage of the opportunity to formalize mechanisms and functions of living systems with precise statements and codes. The result has been decades of complete disarray in gene nomenclature and in the ways of measuring, quantifying and representing biological activities. Perhaps the scientific hooliganism glorified in Watson’s *The Double Helix* (Watson, 1968) is not unrelated to the informal, anti-authoritarian ethos of the scientific community born in that period. While this has not long been a problem, progress in this field and the growing roles of biology and biotechnology in fields beyond the academic environment again raise the need to endow the life sciences with methodologies and languages closer to those of physics than to the descriptive sciences –as biology has been for most of its history. It is in this context that two recent attempts arose to quantify biology, that are likely to completely change our approaches, both methodological and conceptual, to scientific questions and their biotechnological derivatives.

### From molecular biology to systems biology and synthetic biology

The beginning of systems biology was determined by a very practical problem: how to organize and make sense of the avalanche of data derived from the *omics* technologies that began to be applied to biological systems from the end of the 1990s. The sequences of complete genomes were followed by the transcriptomes, proteomes and metabolomes, which led to their corresponding meta-versions (multi-species population data) and to surveys of the same figures in individual cells. Data alone do not automatically become information, however, let alone knowledge; they must be processed with tools not derived from biology, but from computing, information technologies and the physics of complex systems. The *omics* techniques deliver all the data contained in a live biological object, in a more or less cryptic form, that must be deciphered for its comprehension using non-biological instruments (for example, network theory; Barabasi and Oltvai, 2004). This at once offers an opportunity to understand a living system as a whole rather than as its separate parts. But at the same time, this also poses an enormous methodological and epistemological challenge. On the one hand, the analysis of massive data goes beyond being an aid to experimentation to become a genuine source of new information and knowledge. This form of research is unrelated to the great hypothetical-deductive tradition of experimental biology, but very possibly is equally valid. Whole branches of biology that were completely experimental a quarter of a century ago (i.e., microbial ecology) are becoming major platforms for sequence analysis in silico*.* On the other hand, the data must always be projected onto a functional model, which has pushed many systems biologists to specialize in data analysis and mathematical representations, often importing the formalisms of social networks analysis and electronic circuitry. This in turn generates new questions and new research agendas whose objective is to understand the multi-scale complexity of living objects. For the first time, it might be possible to understand the material architecture (the *hardware*) and the operative logic (the *software*) of a living system (Danchin, 2009a, b). To comprehend the whole is to study the whole as such, not only to focus on the details of its components. For this we must draw on abstractions and simplifications typical of physics that help to separate the main components of a system from those that are only spectators or bystanders.

This leads to the next stage: definition of the minimum components a biological system needs to maintain its identity and functions. In the end, full understanding of a system requires not only its analysis but also its synthesis, as the Nobel Laureate in Physics Richard Feynman wrote on his famous posthumous blackboard: *... What I cannot create, I do not understand....*^2^ It was therefore systems biology, with its emphasis on quantification, modelling and the combined use of analysis and synthesis to understand living entities, which set the stage at the beginning of the millennium for the birth and explosive development of synthetic biology as we are witnessing it.

### Looking at living systems with an engineer’s eyes

**Fig. 1:**
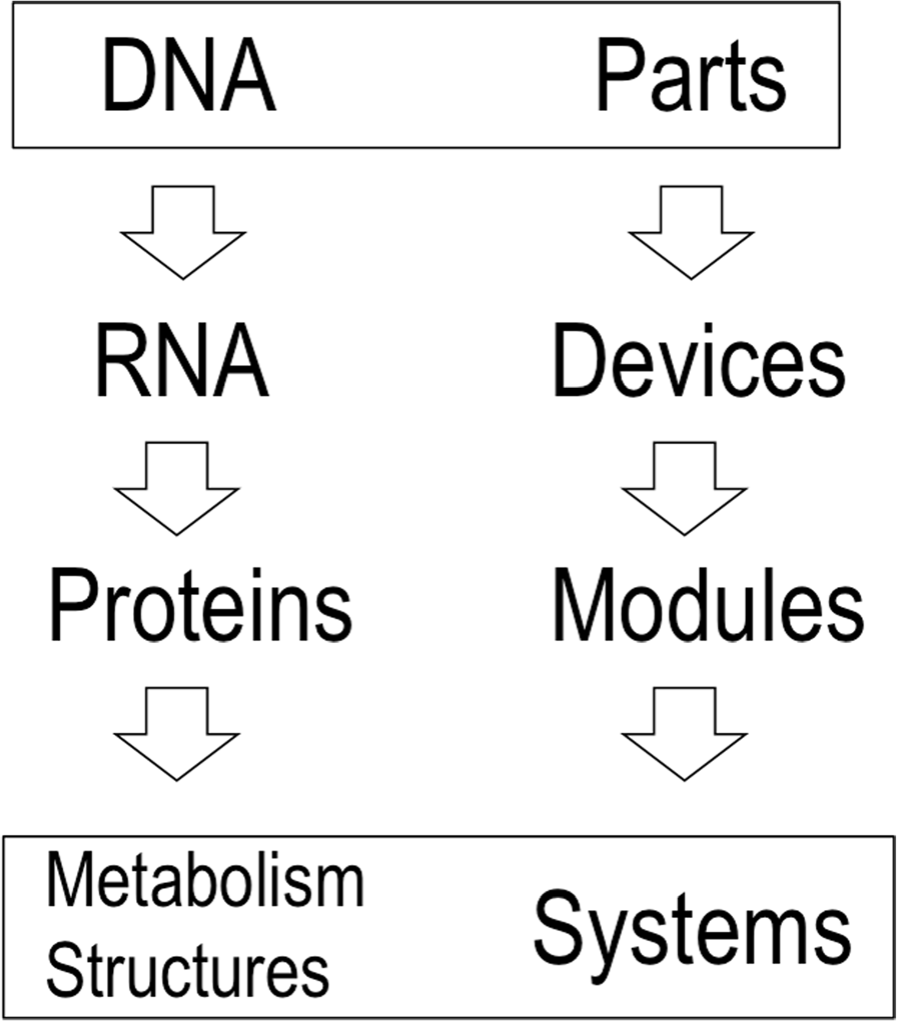
The Central Dogma (CD) of Molecular Biology vs. the core tenet of Synthetic Biology. The original formulation of the CD by Francis Crick (left) states that information is unidirectional, from nucleic acid to protein, never the other way around (Cobb, 2017). Yet, note that information flow is also deployed in the functioning of metabolic networks (as recently proposed: de Lorenzo et al., 2014). On the contrary, Synthetic Biology (right) puts the emphasis on the relational and compositional logic of living systems, both those existing already and those that can be designed in the future

The quantification of biology and the abstractions that are the hallmark of systems biology make a new interpretive framework of living objects almost inevitable. Twentieth century biology used two related hermeneutic frames to understand biological systems. First and foremost is evolutionary theory. Dobzhansky’s well-known assertion that “nothing in biology makes sense except in the light of evolution” defines the *raison d’être* of biological objects as a result of an undirected temporal process of complexity and interactivity in benefit of environmental adaptation and reproductive success. The second interpretive key has come to be known as the central dogma (CD) of molecular biology, that is, the flow of information from DNA ➔ RNA ➔ protein (Fig. 1). These two registers (evolution and CD) allow us to answer the question as to *why* biological systems are as they are and as we know them. But in reality, these same clues tell us little about the operation of the same natural bio-devices and bio-systems, much less about whether they could be mechanistically different from what we see here and now.

Evolution selects functions and their combinations, but not necessarily the specific mechanisms that provide that function. This is seen clearly in the prokaryotic world; the same metabolic or regulatory problem can be solved via different molecular schemes (Cases and de Lorenzo, 2001). The question as to how and why a biological system *works* is thus difficult to answer through only an evolutionary perspective. This is the revolutionary proposal of synthetic biology: to understand the function of living systems, we must consider them as objects endowed with a relational logic between their components not different from those designed by a computational, chemical or electronic engineer (Canton et al., 2008; Endy, 2005). For example, to understand the spatio-temporal distribution of metabolism in a bacterium, knowing its evolutionary origin does not help us much. On the contrary, we would have to ask what a chemical engineer would need to design very small reactors in which thousands of reactions take place simultaneously in space and time. From this question arises the need for compartmentalization (or at least constraints on free diffusion), channelling of substrates and products, protein aging, the problems of toxic waste, and so on (de Lorenzo et al., 2015; de Lorenzo and Danchin, 2008). Only from this engineering perspective can we understand the physicochemical system that is a cell in space and time, putting aside the question as to its origins.

In general, engineered objects must fulfil a function, for which they need instructions (e.g. the software on computers) that are implemented through hardware (the equipment that reads and executes instructions). Traditional molecular biology tends to forget the distinction between function, instructions (software and operating system), and machinery to execute them (hardware). This calls for a qualification of Dobzhansky’s phrase above; evolution mainly selects functions and their combinations, but not the instructions nor the means to read them. A characteristic of living systems is that much of their software/hardware is dedicated to self-replication. As Danchin proposed, in this respect cells can resemble Turing machines able to interpret symbols (chemical, for example –ATGC) printed on a tape (DNA/RNA in this case) according to a table of rules, similar to computers (Danchin, 2009a, b). A machine of this type can be adapted to perform all kinds of operations, including self-assembly. Taking this metaphor to the extreme, cells can be understood as computers that make computers (Danchin, 2009a), not unlike 3D printers that build other 3D printers (Bowyer, 2014).

### Techno-logy vs. techno-nomy

**Fig. 2:**
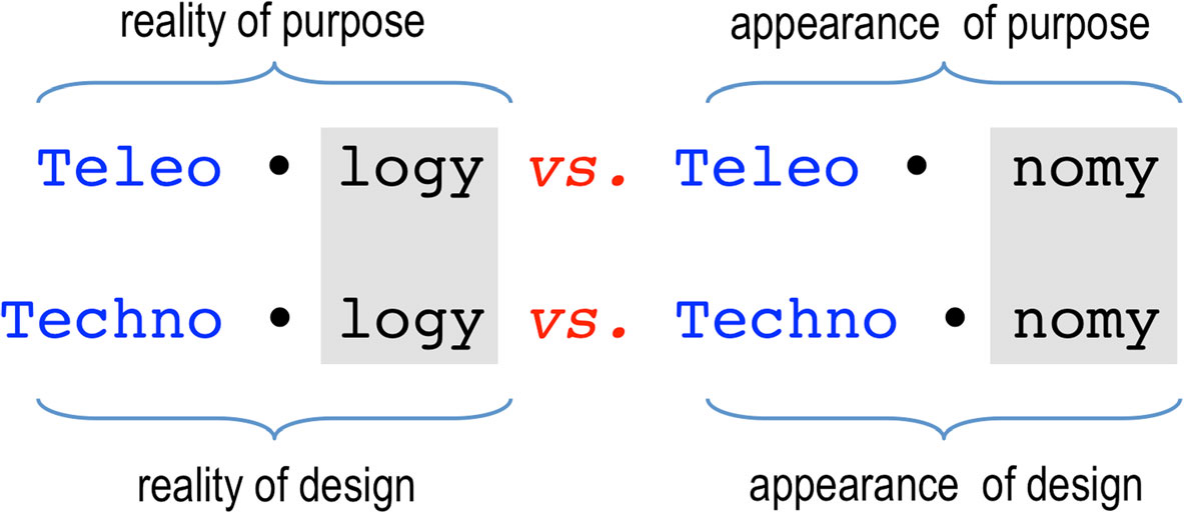
The interpretive frame of synthetic biology for understanding how live systems work. The starting is Monod’s argument (top) on how the *appearance of purpose* in living systems (teleonomy) is a useful tool to understand the logic of biological objects—without accepting metaphysically its reality (teleology). By the same token, the *appearance of design* (what I call *technonomy*) is an invaluable conceptual asset to make sense of the relational composition of live systems that makes them work—without adopting any belief beyond that (e.g. reality of design: technology)

The statement biology-as-engineering nonetheless requires several nuances. First, looking at biological objects as if they were the product of engineering says nothing about the intervention of an engineer. A similar argument was used by Monod in his celebrated discussion on teleology (the *purpose/finality* of biological systems) and teleonomy (the *appearance of purpose/finality* in these systems) in his book *Chance and Necessity* (Monod, 1970). Whereas the former is not within the realm of science, the latter is an extremely useful interpretive frame to understanding why biological systems are as they are and not different. For the same reason, engineering can be adopted as a metaphor and a hermeneutical lens to understanding the logic of biological objects, which is different but perfectly compatible with other explanatory keys that address unlike questions. As sketched in Fig. 2 the creative tension between teleo-logy (purpose) and teleo-nomy (appearance of purpose) we could therefore add a parallel polarity between techno-logy (design) and techno-nomy (appearance of design).

**Fig. 3:**
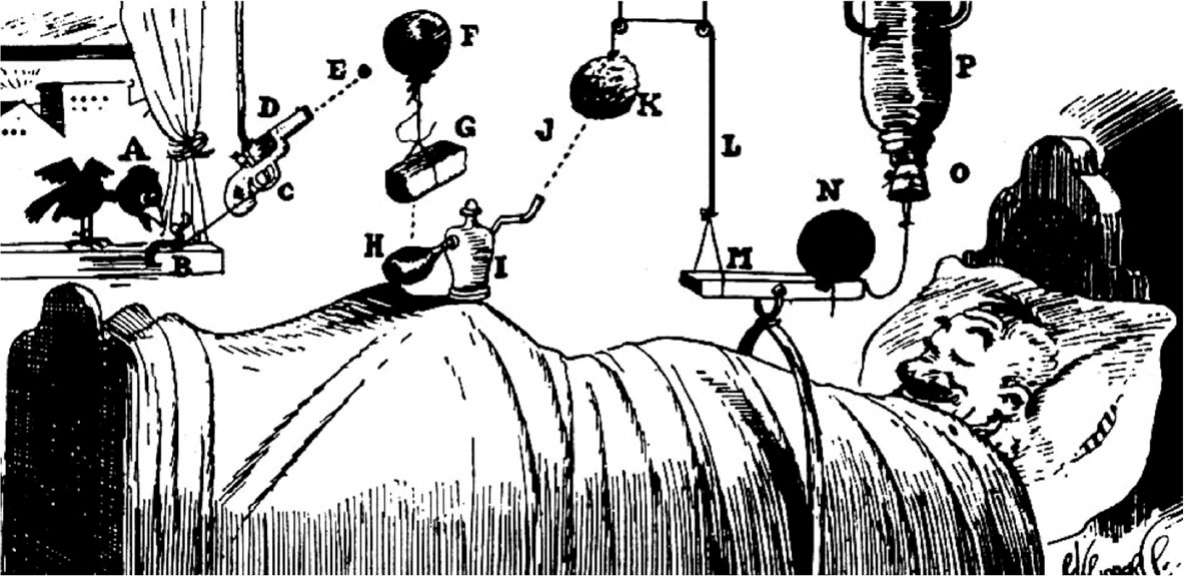
Rube Goldberg machines: simple operations run by complex gadgets. Rube Goldberg (1883–1970) was an American cartoonist popularly known for a series of satirical drawings describing very complicated devices. In the example shown, the simple objective of waking up a gentleman in the morning is disclosed as a chain of 15 events (A-P) run by spare components in which the outcome of each of them triggers the next one. Goldberg’s cartoons make an ironic mockery of unnecessary complexity. Used with permission of RUBE GOLDBERG® (https://www.rubegoldberg.com)

Apart from these somewhat speculative arguments, can we really consider living systems from the viewpoint of an engineer? Building on some of Darwin’s digressions on the coevolution of pollinators and orchids, François Jacob once proposed an insurmountable contrast between engineering and bricolage/pastiche/tinkering as a metaphor for the difference between rational design and biological evolution (Jacob, 1977; Jacob, 1981). Whereas the engineer’s work relies on precise components and tools exactly suited to a predefined project, tinkerers play with odds and ends without knowing what they will produce, and use anything at hand to make some kind of functional object whose usefulness might become apparent later. None of the materials has a precise function originally, and each can be used in several different ways. This view nonetheless appears to say that the structure of living systems has no relational logic comparable to engineering. But taking the tinkering metaphor to an extreme, one could end up in a situation not unlike those of humorous Rube Goldberg machines i.e. intricate designs in which a series of random, spare components that carry out simple operations are somehow linked so that activating one device triggers the next gadget in the sequence (https://www.rubegoldberg.com; Fig. 3). But a candid inspection of data, in particular on the application of synthetic biology approaches for understanding extant biological devices could suggest otherwise. Although different paths can lead to different solutions for design problems, the outcome frequently coincide or converge and one approach can easily help to understand the other. It is not only the wings of planes, birds and bats, but also intricate mechanisms of process control in countless biological objects (Steel et al., 2017).

The same can apply to live systems; although their structure and function cannot be attributed to an engineer, it is very useful to examine them with the perspective and formalisms supplied by engineering. Functions and biological modules that constituted an evolutionary innovation to solve a problem were most successful when they were later assimilated into another context in response to another challenge. For example, when plumage appeared it was merely thermal insulation of dinosaurs, but later became an essential component of bird flight. The analysis of bacterial genomes provides numerous examples of proteins that do something now that turns out to be very different from that for which they originally arose. Functional co-option is in fact very frequent. For instance, extant transcription factors often evolved from enzymes that used as substrates small molecules that later became effectors of the thereby evolved regulators. Also, the same regulatory proteins (e.g. the archetypal CRP regulator of *Escherichia coli*) control expression of entirely different subsets of genes depending on the species where they are (Milanesio et al., 2011). This process, which in evolutionary biology is termed *exaptation*, also has innumerable engineering counterparts: a device invented for a very specific purpose reappears elsewhere with minor modifications and an unexpected function. The system for rapid loading and release of bombs in combat aircraft can be reused for incorporation and replacement of heavy batteries in electric cars (Senor and Singer, 2009). The re-adherable glue borne by Post-it notes was first discarded as a too-weak adhesive until it found a very successful function as a *press and peel* bookmark. An innovation born for one function can triumph when it is assigned another purpose, different and even opposite the original. This scenario appears constantly in biological and in designed systems, reducing what Jacob saw as an insurmountable gap between the two. It will nonetheless be difficult to hold a calm debate on the principle of *techno-nomy* proposed here at a time of confrontation between evolutionism and intelligent design, which became a focal point for heated public debate in the USA and has echoed elsewhere. Coming from a different culture, such a confrontation is not only somewhat farcical but also misleading for tackling the issue of origins vs. functioning of biological objects, as discussed above. Human intelligence is in itself the result of evolution and therefore objects rationally designed by conscious minds might be often indistinguishable from those resulting from a random exploration of a solution space—as they are both obliged to undergo a multi-objective optimization process (see below). Biological evolution and meta-evolution (e.g. conscious intelligent design) may thus deliver the same or similar relational logic in their resulting objects—as otherwise they may not work. Note also that whether evolved or engineered, the outcomes may both be plagued with imperfections and suboptimal solutions that rational design most often produces as well. It may thus be difficult to distinguish whether a given functional item is the result of blind evolution, amateur bricolage or smart design: they all are about finding the same optimal attractors in a solution space through different itineraries. This is something for celebration and one of the most useful contributions of synthetic biology to the scientific research of live systems. Looking at biological phenomena through the lens of engineering has the same potential to transform the field as did looking at biological phenomena through the lens of physics in the post-war period, which led to the birth of molecular biology.

### The modularity of biological systems

A second qualification of the biology-engineering relationship has to do with the modular structure of the objects of study in each case. Any entity designed by an engineer is composed of clearly defined modules, with connectivity between its well-standardized components (which allows re-use in different contexts), with compatible inputs and outputs and a clear hierarchy and three-dimensional arrangement of the various components. This matches the physical and the functional modularity of objects made by the engineers, at least approximately. In contrast, existing biological systems do not at first glance seem to express this coincidence between the physical and the functional. By comparing groups of persistent genes in microbial genomes, the catalogue of functions necessary for a living system has been calculated at about 300–500 (de Lorenzo and Danchin, 2008). A search for specific genes shared by these same genomes nonetheless leads to the surprising conclusion that this number is exactly zero (Acevedo-Rocha et al., 2013). This means that the same functional needs of live systems can be met by very different configurations of genes and molecules.^3^

Another remarkable detail that separates designed objects and biological systems are the physical characteristics of their components: telephones and aircraft are made of *hard* materials, with parts whose three-dimensional structure is clearly defined and has precise connections to neighbouring pieces. Unforeseen interactions often cause problems and cause accidents. In contrast, biological objects are typically composed of *soft* elements, sometimes without clear boundaries and a tendency to interact with one another, which at times leads to the emergence of unanticipated properties. If electrical and industrial engineering consist of cables, tubing and screws, living systems are composed of elastomers, gels and glues. Finally, living systems grow, replicate, and reproduce: properties alien to the rationally engineered objects we know. Does this mean that the principle of modularity we associate with man-made devices is absent in biological systems? Again, the answer is no. The complexity of cells with large genomes and extensive biochemical diversity is misleading in this regard. Analysis of the minimal genomes of endosymbiont bacteria, for example, shows a considerable degree of modularity in the essential functions that allow their existence (Porcar et al., 2013). The biochemical soup that metabolism sometimes appears to be is in fact perfectly modularized, with an organization reminiscent of a chemical factory (de Lorenzo et al., 2015; Huang et al., 2016; Parry et al., 2014). Neither is the idea of self-replicating objects new in engineering, as shown by attempts in the last decade to design three-dimensional printers that print themselves (e.g. the RepRap project: http://reprap.org; Bowyer, 2014).

**Fig. 4:**
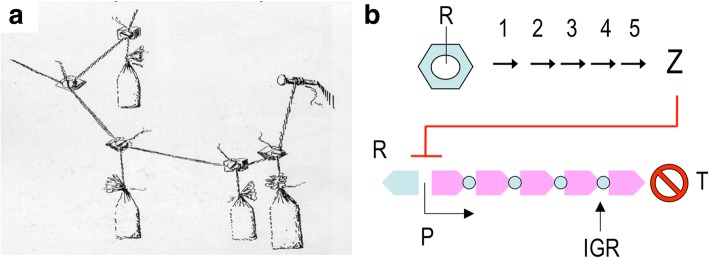
Non-numerical multi-objective optimization. Builders of intricate structures before the scientific era were often faced with the need to play a large number of parameters that were not amenable to the calculation tools available at the time. Architects like Antoni Gaudi (1852–1926) figured out ways to solve the problem by making string models of the building or building parts (**a**) in which weights were hanged at critical places for revealing the effect of local structures on the geometry of the whole object. **b** Uncertainties on the best combination of enzymatic steps (1–5) for converting a substrate into a product (Z) include inter alia reaching a suitable level of transcription (the function of the promoter P and the regulator R) and adequate intergenic regions (IGR) for ensuring the necessary stoichiometry in protein production, as well as mRNA stability and termination (T). Sequence diversification at such regulatory points and selective pressure to increase production of Z allows exploration of the solution space until an optimum is reached

**Fig. 5:**
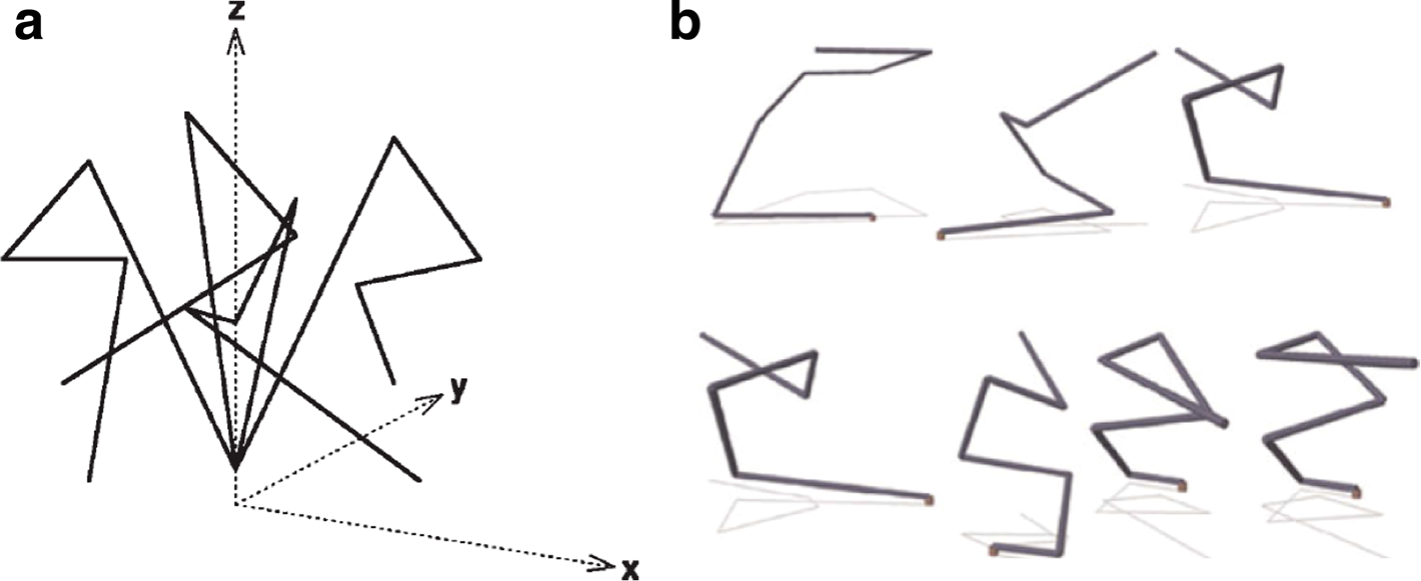
Development of NASA antennas through evolutionary algorithms. **a** Four-wire antenna after application of evolutionary algorithms to a constrained space and multiple specifications (**b**) The sequence of evolved antennas leading up to final object ST5–33.142.7 (Lohn et al., 2008)

It is therefore as possible and productive to use the metaphor and even the formalisms of engineering to understand the function of biological systems as it is to use the biological metaphor to guide the design of new man-made devices. A good part of contemporary engineering is accustomed to randomly exploring the space of solutions to a problem that cannot be resolved by first principles because of the many parameters involved i.e. the challenge of multi-objective optimization. The architect Gaudí, at a time in history that lacked the computational capacity and simulations now common in modern architecture, was able to calculate complex parameters for his buildings by interrogating nature (in his case, gravity in models of ropes and weights) for the optimal configuration of components in his great works (Fig. 4). The interesting thing here is that these solutions to e.g. complex, interconnected catenaries are virtually identical to those found by architects many years later using computation and advanced simulations (Huerta, 2006). It therefore seems that, in engineering as in biology, the space of solutions to an adaptive challenge is neither homogeneous nor it has an infinite number of possible outcomes. Instead, it has attractors (i.e. a set of values towards which a system tends to move regardless of different starting conditions of the system) in which the same outcome can result from directed design or random exploration. One conspicuous case of strategies akin to typical adaptive processes of biology for addressing a multi-objective optimization challenge was the design of antennas ST5–3-10 and ST5-4 W-03 which were deployed in a NASA spacecraft in 2006 (Lohn et al., 2008; Hornby et al., 2011; Fig. 5). The evolutionary algorithms (Coello et al., 2007) adopted to this end delivered objects that were comparable in performance to hand-designed counterparts produced by the contractor for the mission—a clear example of convergence between rational design and evolutionary drives. This shows the value of evolution in shaping optimal devices and vice versa*:* the utility of examining the logic of living systems with the conceptual tools of engineering. It is no surprise that experimental evolution is increasingly merging with synthetic biology. Recent examples include the adaptation of *E. coli* core metabolism to fix carbon with the Calvin cycle through a hemi-autotrophic metabolic mode (Antonovsky et al., 2016; Herz et al., 2017) or the adaptive evolution of a recoded *E. coli* strain (Wannier et al., 2018). But many more examples are in the pipeline: what many call *experimental evolution* or *evolutionary engineering* is in fact an extreme case of multi-objective optimization but involving too large a number of parameters for being rationally tackled—for the time being.

### Genetic engineering: Analogy and methodology

The option for engineering as a key to interpreting the biological phenomena that define synthetic biology has a derivative as fascinating as it is unsettling. It is not just an epistemological question, but also very practical. If a biological system is like an engineered artefact, then we can also dismantle it into a limited set of defined components that we can then recompose to generate a different object based on a rational plan. The result can be an object whose structure and properties differ from those of the original source of its components. To do this we require two things. First, we need the relational and hierarchical abstraction of the new object as a set of parts (the basic units of biological function) that are connected rationally to form devices, and these in turn to generate systems of increasing complexity. At this point, we jump from engineering as a metaphor and analogy (as in genetic engineering) to engineering as a genuine method for constructing biological objects. The narrative^4^ formulation of the central tenet of molecular biology as a process of DNA➔ RNA ➔ protein is thereby replaced by the compositional, quantitative principle of synthetic biology, parts ➔ devices ➔ systems (Fig. 1).

In second place, the parts for engineering new biological systems must be standardized to make them reusable, composable and scalable. In most cases, these parts do not appear this way in their natural situations. We can make a hut with tree trunks just as nature offers them. But to build a house the logs must become beams and panels of precise dimensions that allow the construction of a more complex building (Porcar et al., 2015). By this reasoning, one characteristic of synthetic biology is the effort to start from DNA sequences that determine desirable functions and modify them for use as building blocks (e.g. Bio-Bricks) for new biological objects (Kosuri et al., 2013; Mutalik et al., 2013a; Mutalik et al., 2013b). Based on the existing situation, one can think of modularizing biological functions and components more and more to make them easier to combine, both physically and functionally. This modularizing/standardizing agenda opens up immense prospects for biotechnology: living systems become a source of materials that can generate new objects and properties with little or no similarity to their natural function. A bacterial promoter that, in its native context, controls expression of a tetracycline resistance gene when the cells encounter the antibiotic in the medium is converted by the artistry of synthetic biology into an inverter module (a NOT gate in logic) that can be combined with others to perform calculations and process signals not originally their own (Silva-Rocha and de Lorenzo, 2008). Various bacterial and plant enzymes can be assembled in yeast to give rise to the biosynthetic pathway of an anti-malarial drug (Paddon and Keasling, 2014). Protein anchor sites derived from metazoan signalling pathways have been used in *Escherichia coli* to channel the substrates for a biotransformation of industrial interest (Dueber et al., 2009). And so on, in hundreds of cases in which a biological function is decontextualized using recombinant DNA tools (and more recently by chemical synthesis of DNA sequences) and reused in another situation to do something that nature has not done or invented.

### Simplifying biology to facilitate (re)design

This endeavour faces two major challenges. The physical composition of DNA sequences does not necessarily translate into an integration of the corresponding functions, at least quantitatively. In addition, the parameters associated with the biological parts (promoters, terminators, ribosome binding sites) often change with host genomic context and physiological conditions. Indeed, the problem of context dependence is one of the major limitations in the design of reliable biological devices. Several lines of action have been proposed to remedy this state of affairs.

One of these approaches is to edit the genome and eliminate all complexity not strictly necessary for a given application. In a first phase, the genome can be cleansed of components that cause instability (prophages, insertion sequences, mobile elements), continuing with blocks of genes that, although present and useful in the natural environment (such as the flagellar machinery; Martinez-García et al., 2014), might not be essential in a bioreactor (Posfai et al., 2006; Umenhoffer et al., 2010). This might be followed by elimination of unused metabolic blocks, cell envelope structures and many other genes that might be deemed unnecessary. This approach could ultimately result in a *minimal genome* (Vickers, 2016) and thus simplify the molecular context of any device that could be implanted in it. Yet, attempts to reduce the genome of model bacteria such as *E. coli* have in fact failed to exceed 20–30% (Csorgo et al., 2016). Apart from the elimination of possible essential genes, deletion of large chromosome segments could alter its architecture within the cell, making it unviable.

An alternative is to proceed in exactly the opposite direction, starting with bacteria whose genome is already very small, such as *Mycoplasma* or endosymbionts like *Buchnera* (Roeland et al., 2003). In these cases, nature itself has made the reduction. Although this can be a good approach in principle, that a system has fewer components does not mean that the outcome will be simpler. Reduced compositional complexity is compensated by an increase in relational complexity; chromosomes with fewer genes give rise to cells that are much more dependent on interaction with the environment. Even so, some bacteria with small genomes (such as *Mycoplasma*) have become models of reference in synthetic biology, particularly because their chromosome size permits complete chemical synthesis, as done by the Venter group (Hutchison et al., 2016) recently extended towards yeast (Kannan and Gibson, 2017; Richardson et al., 2017). This enables implementation of the scenario above, considering bacteria and other biological systems as computers for which software (DNA) can be written and applied by existing molecular machinery. This is the direction of Venter’s futuristic proposals for a digital biological converter (Boles et al., 2017; Corbyn, 2013).

### Orthogonalization

But simplifying the genome and even rewriting it completely does not solve all the problems. As mentioned above, the operation of biological parts, especially quantitative, is subject to varying degrees of influence at various contextual levels —from interference from nearby sequences to general and environmental effects. To the benefit of evolution, but to the irritation of bioengineers, biological materials (proteins, polymers, small molecules) tend to interact with their molecular neighbours in often unpredictable ways. In biology, 2 + 2 are not always 4, because any new combination is subject to the emergence of new properties, negative or positive, that cannot be predicted from the qualities of the components of the sum, at least not with the degree of knowledge we have in most cases. A situation familiar to any biotechnologist is uncertainty regarding the efficiency of heterologous expression systems for genes of industrial interest. The combination of a strong promoter with a strong translation initiation signal should in principle lead to strong expression (transcription + translation) of the gene of interest. This is often the case, but on occasion the opposite is true (Kosuri et al., 2013). Why? Very often, the transcript 5′ end forms unexpected secondary structures with sequences downstream of the gene, which generates instability in the mRNA or prevents translation (Espah-Borujeni et al., 2017).

A possible remedy for these situations is the so-called orthogonalization of the system’s components. Two systems are mutually orthogonal if they do not influence each other. It is conceivable to start from a very connected biological component or module to produce a variant that retains only the desired connectivity, thus facilitating its use for new biological designs. Nature itself offers cases of orthogonal parts, typically in promiscuous mobile elements and bacteriophages (e.g., T7 phage RNA polymerase). But great progress has also been made in developing alternative genetic codes and orthogonal ribosomes able to decipher them. Perhaps in the not too distant future we can have biological entities with a genome that encrypts information with a distinct genetic code (even using non-natural bases; Malyshev et al., 2014) expressed with alternative polymerases and whose messages are translated by orthogonal ribosomes. The resulting living object would be so far removed from those we know that it could not interact in any way with natural biological systems, ensuring its containment and the safety of its biotechnological use (Schmidt and de Lorenzo, 2012, 2016). In any case, the pursuit of orthogonal functional modules or even entire organisms may not be the ultimate way to go for designing biological systems. But they can be a useful interim solution in the way towards an authentic biological engineering until we know more about the rules that make natural living objects to work as they do.

### Stop evolution?

The challenges synthetic biology faces to become a true branch of engineering do not end with the points we have discussed so far. The most important remains: to ensure that any designed device or living object maintains its properties over time and does not yield to noise and mutations, or develop new properties. Even if we optimize the layout of a biological circuit or a complete system, it is inevitable that with time, the DNA that determines it will mutate (much more likely if there is environmental stress), leading ultimately to collapse of the entity. The scientific and biotechnological literature holds many examples of recombinant microorganisms designed for a specific function that, after some time, no longer carry out the desired genetic program due to accumulated mutations (Rugbjerg et al., 2018a). The obvious question is whether we can stably force natural systems to do for our benefit something they do not do habitually. The predominant strategy for addressing this challenge is to penalize (through ad hoc genetic circuits) mutations that lead to failure to achieve the objectives, for example by inducing elimination of undesired mutants (Rugbjerg et al., 2018b). But like any genetic construct, conditional lethality circuits are also subject to mutations that render them inefficient. This challenge has been broached, but remains unsolved. The proposals range from refactoring the information-bearing molecules (from DNA to partially or totally artificial polymers) to a complete change in the information medium, from being encrypted by coding molecules other than DNA to being determined by lipid composition. This is an authentic bottleneck that must be addressed so that synthetic biology can fulfil its promises.^5^

## Conclusions and outlook

The research agenda of synthetic biology can easily be deduced from what has been said so far. Besides evolution, the main objective obstacle to engineering biological systems is the influence of the multi-scale context in the function of individual components of any living entity. Reduction of genomic complexity, the orthogonalization of parts and devices to be combined, and the elimination of mutants that lose the program implanted in them are obvious paths to follow, but that is not all. To advance in the design of these objects, we must answer some fundamental biological questions. The first is to clarify the relationships between cell metabolism, growth, division and proliferation, starting with the simplest, bacteria. As discussed earlier, unlike man-made devices, living systems grow, which adds extraordinary complexity for their predictable design. One would ideally like to have cells that perform the functions for which they are designed, but do not grow. This raises fascinating research challenges, since any program implemented with material components ages and generates errors. In biological systems, the means for repair is to re-create them in the copies generated during growth. Given that the evolutionary program of living beings is reproductive success, can we uncouple growth from the rest of biological function without seriously altering the cells? This is a problem that molecular biology will be hard put to answer alone, as it has derivatives in information theory, nanomaterial resistance, and mechanical systems engineering.

Another limitation yet to be explored in detail is the influence of metabolism on the flow of gene expression. The abstractions of the circuits and modules to be implanted in biological systems often neglect the fact that they act in a complex, highly reactive chemical environment with its own logic. That which in synthetic biology jargon is termed the *chassis* is made up not only of a more or less intricate genome, but also of a scenario of great molecular complexity that we barely grasp. How we understand this multi-scale, multi-molecular complexity will determine to a large extent whether the developments of synthetic biology become robust technologies or come to nothing.

At best, once one has reliable components at hand to build a complex object (like a Meccano or Lego set), it is up to the user’s imagination to produce all kinds of articles and materials, from biofuels, biocatalysts and new therapeutic agents to different forms of computation, bioplastics and intelligent fibers or biosensors for a variety of molecules. It is precisely through imagination and creativity that our scientific and technical community can make its greatest contributions to the field. It is thus necessary to foster the life sciences-engineering interface in university academic programs and to promote degrees that integrate fundamental biology and engineering principles in equal part. At the moment, many molecular biologists claim that engineers know little or no biology, which makes it difficult to interact with them. The engineers in turn see many biologists as lacking the quantitative talent and mathematical training needed to design systems that really work. Overcoming this cultural barrier is possibly the greatest challenge, and we depend on its solution to be actors and not mere spectators of the new type of bioscience and bioindustry that will develop in coming decades. Yet, it cannot be insisted enough that, robust as it has been argued throughout this paper, the engineering metaphor applied to Biology—whether synthetic or natural— is still a metaphor and thus unable to capture reality in all its entirety. We should not turn a blind eye to the fact that embracing engineering as the ultimate frame for addressing biological systems has been seriously criticised (Boudry and Pigliucci, 2013; Nicholson, 2013; Pauwels, 2013; de Lorenzo, 2011). Even the text above resorts to metaphoric terms (genome editing/writing, circuit, hardware, software, device etc.) that may not be entirely warranted in the corresponding context. This awareness is of essence for guiding responsible research in the field and adopting a healthy relativisation of any conceptual frame in Life Science research. Ultimately, as happens with scientific hypotheses also, all metaphors may be ultimately wrong, but some of them are surely (very) useful.

## Footnotes

1 In this context, *quantitative* means something that can be measured and given specific values—whether numerical in the case of metrology or symbolic/virtual in case of e.g. computer simulations, geometrical shapes or other mathematical devices.

2 The complementary statement may not be true, though. We can create many objects that work without understanding why they do. Creation is thus a necessary —but not sufficient— condition for understanding.

3 Note that there may be a methodological caveat in the comparative genomics approach to reconstruct genes shared between divergent organisms. The possibility exists that earlier structural similarities have been erased by time. In that case, homology cannot be easily established quantifying similarity in primary sequences and, in occasions, could be traced instead to three-dimensional shapes. Accumulation of neutral mutations can obscure primary structures maintaining form and function. Structural plasticity and functional promiscuity can also be a source of apparent dissimilarity between biological devices originated in the same precursor.

4 The term *narrative* includes *descriptive* but it also embodies some type of *story-telling*. If I say *the formula of carbon dioxide is CO*_*2*_, that is *descriptive*. If I say *Enzyme X has evolved from enzyme B,* I enter also some type of *narrative*. I thus argue that the Central Dogma and other principles in Biology goes well beyond being merely descriptive.

5 Evolution enabled by genetic diversification can be slowed down down by mutating components of the SOS response to DNA damage and eliminating genomic instability determinants e.g. insertion sequences and other mobile elements. But could it be stopped altogether? In the Author’s view, this would be the ultimate multi-objective optimization challenge for Synthetic Biology. If such a solution exists, it could be found with a directed evolution system that progresses itself towards not-evolving. Nature offers examples of what appears to be evolution towards no-evolution e.g. in cystic fibrosis infections (Marvig et al., 2015; La Rosa et al., 2018) and endosymbionts (Roeland et al., 2003).
